# Predictors of Long COVID Among Symptomatic US Adults Testing Positive for SARS-CoV-2 at a National Retail Pharmacy

**DOI:** 10.3390/healthcare12232321

**Published:** 2024-11-21

**Authors:** Xiaowu Sun, Manuela Di Fusco, Laura L. Lupton, Alon Yehoshua, Mary B. Alvarez, Kristen E. Allen, Laura Puzniak, Santiago M. C. Lopez, Joseph C. Cappelleri

**Affiliations:** 1 CVS Health, Woonsocket, RI 02895, USA; luptonl@cvshealth.com; 2 Pfizer Inc., New York, NY 10001, USA; manuela.difusco@pfizer.com (M.D.F.); alon.yehoshua@pfizer.com (A.Y.); mary.alvarez@pfizer.com (M.B.A.); kristen.allen@pfizer.com (K.E.A.); laura.puzniak@pfizer.com (L.P.); santiago.lopez@pfizer.com (S.M.C.L.); joseph.c.cappelleri@pfizer.com (J.C.C.)

**Keywords:** SARS-CoV-2, long COVID, predictive model

## Abstract

Background: Long COVID remains a significant public health concern. This study investigated risk factors for long COVID in outpatient settings. Methods: A US-based prospective survey study (clinicaltrials.gov NCT05160636) was conducted in 2022 and replicated in 2023. Symptomatic adults testing positive for SARS-CoV-2 at CVS Pharmacies were recruited. CDC-based long COVID symptoms were collected at Week 4, Month 3, and Month 6 following SARS-CoV-2 testing. Logistic regression was used to develop a predictive model for long COVID using data from the 2022 cohort. The model was validated with data from the 2023 cohort. Model performance was evaluated with c-statistics. Results: Patients characteristics were generally similar between the 2022 (N = 328) and 2023 (N = 505) cohorts. The prevalence of long COVID defined as ≥3 symptoms at Month 6 was 35.0% and 18.2%, respectively. The risk factors associated with long COVID were older age, female sex, lack of up-to-date vaccination, number of acute symptoms on the day of SARS-CoV-2 testing, increase in symptoms at Week 1, underlying comorbidities and asthma/chronic lung disease. The c-statistic was 0.79, denoting good predictive power. Conclusions: A predictive model for long COVID was developed for an outpatient setting. This research could help differentiate at-risk groups and target interventions.

## 1. Introduction

SARS-CoV-2 infections remain widespread and continue to cause substantial public health burdens [1]. Symptoms of acute COVID-19 disease vary widely and may include respiratory symptoms (such as cough, congestion, shortness of breath), constitutional symptoms (such as fever, chills, fatigue), gastrointestinal symptoms (vomiting, diarrhea) and generalized symptoms such as muscle pain, among others [2]. Although most patients fully recover from acute COVID-19 disease, some may go on to develop long COVID [3].

Long COVID, also known as post-acute sequelae of SARS-CoV-2 infection, is a complex condition characterized by symptoms and conditions that persist or newly present after an initial SARS-CoV-2 infection or reinfection [4,5,6]. As with acute COVID-19, symptoms vary widely across multiple body systems and may reflect a continuation of acute COVID-19 symptoms or present as new manifestations such as extreme fatigue, cardiac symptoms (such as palpitations), or neurological symptoms (such as difficulty concentrating), among others [3]. These symptoms can be persistent and debilitating, significantly impacting a person’s quality of life [7,8,9]. According to the U.S. Census Bureau’s Household Pulse Survey (HPS), during 28 May–24 June 2024, 18.4% of all adults or 31.0% of adults with a prior history of COVID-19 report having ever experienced long COVID, and 5.3% of all adults or 8.9% of adults with a prior history of COVID-19 report experiencing long COVID currently [10]. In the United States, the economic burden of COVID-19 is estimated to be trillions of USD, driven by diminished quality of life, lost earnings, and higher spending on medical care [11].

Since January 2022, the Omicron variant and its various sub-lineages have remained predominant in the U.S. [12]. Compared with earlier variants, Omicron variants have a higher transmission rate [13] but a lower risk of severe outcomes [13,14]. Research to characterize long COVID and its risk factors is ongoing [4,15,16,17,18], though research in outpatient settings during Omicron predominance is still scarce [19].

This study focused on prospective outpatient reported data to create a risk assessment with predictive value for long COVID. This study leverages previously described study cohorts [8,9] to explore the risk factors for long COVID in outpatient symptomatic adults with lab-confirmed SAR-CoV-2 infection during Omicron variant predominance.

## 2. Data and Methods

### 2.1. Study Design

A nationwide prospective patient-reported outcomes survey on the impact of COVID-19 (clinicaltrials.gov NCT05160636) was conducted in 2022 and replicated in 2023 [20,21]. Symptomatic adults (age 18 years or older) testing positive for SARS-CoV-2 by polymerase chain reaction or rapid antigen test at CVS Pharmacy stores were invited to participate between 31 January and 30 April 2022 (2022 study wave), and between 2 March and 18 May 2023 (2023 study wave). Patients were excluded from the 2022 study if they received a mixed primary vaccine series or a series other than BNT162b2, or from the 2023 study if a non-BNT162b2 vaccine was received <12 months prior to enrollment. Long COVID symptoms were collected at Week 4, Month 3, and Month 6 following a positive test.

### 2.2. Baseline Characteristics

The baseline characteristics of study participants’ demographics, comorbidities (including asthma or chronic lung disease, cirrhosis of the liver, immunocompromised conditions or weakened immune system, diabetes, heart conditions or hypertension, overweight or obesity), living and work settings, and COVID vaccination history were obtained via a pre-test questionnaire. Subjects were considered up to date with COVID vaccination if they reported receipt of at least one dose after the primary series of the BNT162b2 vaccine in the 2022 study or the BNT162b2 BA.4/5 bivalent mRNA vaccine in the 2023 study. The zip-code-based social vulnerability index (SVI) was applied with ranges from 0 to 1, where higher values represent more vulnerable communities [22]. Antiviral treatment status was not assessed during the 2022 study and was self-reported by patients during the 2023 study.

### 2.3. Acute COVID Symptoms

The presence of acute symptoms was assessed via a questionnaire that included the 12 symptoms based on the Centers for Disease Control and Prevention (CDC) list [2] at the time of study initiation. The questionnaire was administered at the time of SARS-CoV-2 testing and in Week 1 in both the 2022 and 2023 study waves; an additional assessment at Week 2 was added in the 2023 study wave. These symptoms included systemic (fever, chills, muscle or body aches, headaches, fatigue), respiratory (shortness of breath or difficulty breathing, cough, sore throat, new/recent loss of taste or smell), and gastrointestinal (GI) (nausea or vomiting, diarrhea) [2].

### 2.4. Long COVID Symptoms

In the 2022 study, the presence of long COVID symptoms was assessed via a 20-symptom questionnaire based on the CDC list that was current at the time of data collection [6]. In the 2023 study, the presence of long COVID was assessed via a 30-symptom questionnaire, augmenting the CDC list that was current at the time of data collection. While the symptoms broadly overlapped between the two studies, the 2023 study relied on a larger symptom set.

In both studies, the questionnaire was administered at Week 4, Month 3, and Month 6 after testing positive for SARS-CoV-2. The list of symptoms in the 2022 study included general symptoms (tiredness or fatigue, symptoms that worsen after physical or mental activities, fever, general pain/discomfort); respiratory and cardiac symptoms (difficulty breathing or shortness of breath, cough, chest or stomach pain, fast-beating or pounding heart (also known as heart palpitations); neurological symptoms (change in smell or taste, headache, dizziness on standing (lightheadedness); difficulty thinking or concentrating (brain fog, pins-and-needles feeling, sleep problems, mood changes, memory loss); and digestive symptoms and other symptoms (diarrhea, joint or muscle pain, rash, changes in menstrual cycles). The analysis was limited to the original list of 20 symptoms from the 2022 study, excluding the additional symptoms from the 2023 study, to allow for a uniform comparison over time.

As previously described [8,9], long COVID was defined as reporting ≥3 symptoms at the time of each assessment (Week 4, Months 3 and 6), which is consistent with the methodology used in similar survey-based studies [23,24]. Alternative case definitions of ≥2 symptoms and ≥4 symptoms were used for sensitivity analysis.

### 2.5. Statistical Analysis

Descriptive statistics were used to summarize participant characteristics and long COVID symptoms. Mean and standard deviation (SD) were used for continuous variables, and frequency and percentages were used for categorical variables.

The predictive model was developed for long COVID based on the 2022 study. Stepwise logistic regression adding/removing one variable at a time was conducted with all candidates, including sociodemographic variables, comorbid conditions, number of acute symptoms on the day of SARS-CoV-2 testing, Week 1 change in number of acute symptoms, individual acute symptoms during testing, and vaccination status.

An initial significance level of 0.15 was set for a variable to enter and stay in the model, while variables below the threshold were removed. The significance level was then adjusted to 0.05 in our final model.

The model was further modified to be more reliable and parsimonious without sacrificing performance. The risk score was calculated as the summation of variables for the risk factors multiplied by their corresponding coefficients for both the 2022 study and the 2023 study. The predictive power of the model was evaluated using c-statistics. The value of c-statistic ranges from 0.5 to 1, with a larger value denoting a more predictive model. Values ≥ 0.7 are considered acceptable and indicative of good predictive power [25]. All available data were analyzed, and no imputation for missing values was attempted. All analyses were conducted with SAS version 9.4 (SAS Institute, Cary, NC, USA).

## 3. Results

In the 2022 study and 2023 study, 39,889 and 21,113 qualified patients were contacted, respectively. Of those, 676 and 729 consented [20,21]. The long COVID study population consisted of 328 subjects from the 2022 study and 505 subjects from the 2023 study, with 87 (26.5%) and 260 (51.5%), respectively, considered up to date with vaccination recommendations. The mean age was 42.0 years (in 2022) and 46.3 years (in 2023), and more than 70% of the enrolled subjects were female. The mean number of acute symptoms on the day of SARS-CoV-2 testing was 5.39 (in 2022) and 5.31 (in 2023), with subsequent values of 2.76 and 2.59 at Week 1 (Table 1 and Table S1). Patient characteristics and the acute symptoms of each study were published previously [8,9].

The frequency of long COVID assessed as three or more long-term symptoms was 39.6%, 37.3%, and 35.0% at Week 4, Month 3, and Month 6 in the 2022 study, while it was 25.7%, 20.0%, and 18.2% at Week 4, Month 3, and Month 6 in the 2023 study (Table 2 and Figure S1).

Ten variables were selected by using a stepwise selection approach. The initial model included age as a continuous variable, female sex, lack of up-to-date vaccination, asthma or chronic lung disease, number of comorbidities, number of acute symptoms on the day of SARS-CoV-2 testing, an increase in number of acute symptoms from the day of SARS-CoV-2 testing to Week 1, and three acute symptoms (‘nausea or vomiting’, ‘cough’, and ‘congestion’) on the day of SARS-CoV-2 testing. The c-statistic of the model was 0.80 (95% CI: 0.77, 0.83) (Table S2).

The model was then revised as follows. Age was categorized to allow for a flexible relationship. ‘Nausea or vomiting’ demonstrated directional changes when the model was refit with 2023 wave data. Cough and congestion commonly co-presented and had opposite effects on long COVID, nearly canceling each other out. This is difficult to interpret clinically, and as it did not sacrifice model fitting, we chose to eliminate these variables. At every step, the model was refit, and the significance of variables was reassessed. The final model is presented in Table 3. The c-statistic of the model was 0.79 (95% CI: 0.76, 0.82).

The model showed that subjects of older age, of female gender, with a lack of up-to-date vaccination, higher acute symptom frequency and persistency, and underlying comorbidities (especially respiratory conditions) had higher odds of long COVID (Table 3). Compared with young adults 18–29 years, subjects of 50–64 years had the highest risk of long COVID, with an odds ratio (OR) of 1.72. The risk of long COVID was associated with female sex and lack of up-to-date vaccination, with an OR of 2.06 and 1.72, respectively. The OR of long COVID was 2.61 in subjects with asthma or chronic lung disease (OR = 1.96 × 1.33). A higher risk of long COVID was associated with more symptoms on the day of SARS-CoV-2 testing and persistent symptoms in Week 1. Each additional symptom reported on the day of testing was associated with an OR of 1.72 for long COVID. On average, subjects reported 5.39 symptoms on the day of SARS-CoV-2 testing. For two patients with five symptoms on the day of testing, if one patient’s symptoms resolved at Week 1 and the other patient’s illness was persistent with no change in the number of symptoms, then the patient with persistent symptoms would have an OR of 7.4 of long COVID [OR = exp (5 × 0.40)] compared to the patient whose symptoms resolved.

Risk scores were calculated for each subject using the model in Table 3. The logistic model with this risk score had a c-statistic of 0.76 (0.73, 0.79) using 2023 wave data. Similarly, logistic models with long COVID defined as ≥2 and ≥4 long symptoms all had c-statistics greater than 0.7, with a range from 0.73 to 0.83 (Table 4).

## 4. Discussion

Utilizing two nationwide patient-reported outcome survey waves that evaluated long COVID symptoms throughout six months following SARS-CoV-2 infection, we developed and validated a prediction model showing that certain clinical and demographic characteristics were associated with an increased risk of long COVID. The model included age, gender, chronic lung disease, number of acute symptoms and change in number of acute symptoms during the first week after testing. We found that the risk of long COVID was higher for participants of older age (especially 50–64), of female gender, with chronic respiratory conditions, and with higher symptom frequency and persistence during the acute disease stage. These results were consistent across the two studies with different variant predominance, and across all three long COVID definitions. The resulting predictors are easily captured risk factors that can be used to assess the risk of long COVID in an outpatient setting.

Long COVID is recognized as a major public health threat by public health institutions [26]. Existing studies exploring the predictors of long COVID-19 used data either from earlier years with a focus on hospitalized patients or from retrospective or cross-sectional surveys [15,16,17,18,19]. Particularly novel in this study is the use of two waves of longitudinal surveys over two consecutive years from the community setting. This provides updated insights on relevant factors associated with long COVID-19.

Prior studies showed that long COVID-related risk factors include demographics (female biological gender, older age), poor health conditions (high BMI, smoking, presence of comorbidity), acute COVID-19 conditions (disease severity, duration, previous inpatient or ICU admission due to COVID-19), and vaccination status (not vaccinated, not up-to-date vaccination) [27,28,29]. Earlier SARS-CoV-2 variants have also been associated with a greater risk of developing long COVID [28,29].

Since the June 2022 Phase 3.5 of the Household Pulse Survey, the question for long COVID (‘Did you have any symptoms lasting 3 months or longer that you did not have prior to having coronavirus or COVID-19?’) has been added to the survey [30]. Females reported about 60% more long COVID than males. The incidence of long COVID peaked at 50–59 years, then decreased in older age groups. Despite the different definitions of long COVID, these findings were generally consistent with our study.

Acute disease severity is associated with the risk of long COVID [31,32]. In the current study, the subjective severity of acute symptoms or the overall condition was not directly collected. Instead, the number of symptoms was used as a surrogate for the severity of acute illness. The premise is that patients with severe illness during the acute phase of infection are likely to report a higher number of symptoms. In addition, worsened health-related quality of life, greater loss of work productivity, and activity impairment were found to be significantly associated with the number of acute symptoms [20,21].

Staying up to date with COVID-19 vaccination is a core strategy for protecting against long COVID [6]. Our study found that COVID-19 vaccination has a protective effect against long COVID. Participants who were up to date with BNT162b2 vaccination had a significantly lower risk of long COVID compared to those that were not up to date or were unvaccinated. This was observed consistently in both the 2022 and the 2023 study data. We previously reported that, in the 2022 study, during the Omicron variant lineages BA.1.1- and BA.2-predominant period, individuals boosted with the original monovalent BNT162b2 vaccine reported a lower incidence of long COVID compared with unvaccinated and fully vaccinated (primed) subjects [8].

In the 2023 study, during the Omicron XBB.1.5-predominant period, subjects vaccinated with the BNT162b2 BA.4/5 bivalent vaccine experienced a reduced risk of acute COVID-19, with significantly greater protection against long COVID than subjects unvaccinated or not up to date with vaccination [9]. Our results are in line with findings from meta-analyses showing that COVID-19 vaccination significantly lowers the risk of developing long COVID [27,28,29]. Because the protective effect of vaccination wanes over time and new variants may exhibit immune escape [33], the effect of vaccination status on long COVID should be monitored over time.

The current study is subject to several limitations. Firstly, the analysis may be subject to bias associated with self-reported data. For example, there might be uncertainty and recall bias in the measures of exposure to SARS-CoV-2 that were available, such as ‘previously testing positive’.

Secondly, there might be non-response bias and selection bias due to differences between the population for long COVID analysis and the outreach population. The analysis population was female over-represented and healthier [20,21]. However, these sociodemographic factors were controlled for in the analysis and produced results consistent with the observed findings [20,21]. With regards to non-response bias, little relationship was found with survey response rate [34,35]; nevertheless, this may not be generalizable to self-reported HRQoL in the current setting.

Thirdly, there might be selection bias, since enrollment was voluntary after a positive SARS-CoV-2 test. Individuals presenting with more severe symptoms may not enroll in a survey or may seek higher levels of care, while individuals with very mild disease or with poor access to healthcare may not seek testing or treatment. As a result, the generalizability of the tool would need to be evaluated outside of an outpatient setting.

Fourthly, the definition of long COVID varies across studies [36], and there is no universal definition. In the current study, long COVID was defined based on number of symptoms reported at each survey time point. Results were robust across the cut-off values used to define long COVID.

Fifthly, because of the heterogeneity in the mechanism of developing long COVID and associated symptoms, different risk factors may be found to be associated with different clusters of symptoms [4,37,38,39]. However, a previous study found that symptom phenotypes found via latent class analysis differed in magnitude of burden with respect to number of symptoms rather than clusters of symptoms [40].

Sixthly, antiviral treatment is one of the core strategies for preventing severe outcomes from COVID-19 that, in turn, may help to prevent long COVID, especially for those patients with risk factors for severe illness [6]. This study did not collect antiviral use in the 2022 survey wave; hence, we did not have appropriate data to estimate the contribution of antiviral use.

Lastly, future studies could use a longer follow-up period beyond six months to capture the burden of long COVID and generate additional insights on long-term vaccine effectiveness. Also, the study relied on the use of diagnostic tests to detect SARS-CoV-2 (PCR and antigen), and genomic characterization was not available. Therefore, the specific sub-lineages could not be captured, but all cases were collected during a period of Omicron predominance, as identified through the CDC variant tracker. Because of these limitations, the generalizability of findings from this study to other settings in terms of different SARS-CoV-2 variants, populations, long COVID definitions, etc., may be limited.

## 5. Conclusions

This study showed that a significant portion of patients with mild COVID-19 disease experienced long COVID symptoms up to 6 months after an infection with an Omicron variant of SARS-CoV-2. The predictive model showed that, across three case definitions and two studies, certain clinical and demographic characteristics were associated with an increased risk of long COVID. The long COVID symptoms were more likely in participants of older age, of female gender, with a lack of up-to-date COVID-19 vaccination, with a higher acute symptom burden, and with underlying comorbidities, especially respiratory conditions. This model captures easily identifiable risk factors in an outpatient setting. Understanding who may be at risk of developing long COVID has implications in the acute management of patients and may inform the targeting of early interventions. That this model performed equally well across two COVID-19 seasons with differences in the predominant SARS-CoV-2 variant demonstrates its potential utility across the spectrum of outpatient care in a continually evolving disease landscape.

## Figures and Tables

**Table 1:** Baseline characteristics and acute symptoms.

	Year 2022 (n = 328)	Year 2023 (n = 505)	*p*-Value ^a^
Vaccination status, n (%)			<0.001
Unvaccinated or no up-to-date vaccination	282 (73.5)	245 (48.5)	
Up-to-date vaccination (BNT162b2 monovalent boosted in 2022; or BA.4/BA.5 Bivalent BNT162b2 in 2023)	87 (26.5)	260 (51.5)	
Age, years, mean (SD)	42.0 (14.5)	46.3 (15.5)	<0.001
Age groups, years, n (%)			
18–29	73 (22.3)	76 (15.0)	<0.001
30–49	160 (48.8)	220 (43.6)	
50–64	67 (20.4)	125 (24.8)	
≥65	28 (8.5)	84 (16.7)	
Female, n (%)	242 (73.8)	357 (70.7)	0.428
Race/Ethnicity, n (%)			0.005
White or Caucasian (not Hispanic or Latino)	234 (71.3)	305 (60.4)	
Black or African American	13 (4.0)	40 (7.9)	
Hispanic	44 (13.4)	73 (14.5)	
Asian	16 (4.9)	49 (9.7)	
Other	21 (6.4)	38 (7.5)	
U.S. region, n (%)			<0.001
Northeast	41 (12.5)	68 (13.5)	
South	188 (57.3)	201 (39.8)	
Midwest	63 (19.2)	113 (22.4)	
West	36 (11.0)	123 (24.4)	
Social vulnerability index, mean (SD)	0.43 (0.22)	0.44 (0.23)	0.799
Previously testing positive	121 (44.6%)	204 (43.0%)	0.670
Work in high-risk setting	33 (10.1%)	64 (12.7%)	0.243
Live in high-risk setting	16 (4.9%)	25 (5.0%)	0.962
Work in healthcare	37 (11.3%)	75 (14.9%)	0.140
Self-reported comorbidities			
Number of comorbidities, mean (SD)	0.35 (0.65)	0.38 (0.75)	0.533
≥1 comorbidity	87 (26.5%)	127 (25.1%)	0.657
Antiviral treatment	-	122 (24.2%)	
Number of symptoms on the day of SARS-CoV-2 testing, mean (SD)	5.39 (2.57)	5.31 (2.30)	0.651
Number of symptoms at Week 1 after testing, mean (SD)	2.76 (2.20)	2.59 (1.77)	0.212
Change in number of symptoms from the day of SARS-CoV-2 testing to Week 1, mean (SD)	−2.63 (2.73)	−2.73 (2.42)	0.597

^a^ *p* values were based on two-sample *t*-tests for continuous variables and on chi-square tests for categorical variables or Fisher’s exact tests when frequency was less than 5.

**Table 2:** Summary of long-term symptoms.

	Week 4	Month 3	Month 6
2022 study			
N	328	292	260
mean (SD)	3.1 (3.6)	2.7 (3.5)	2.7 (3.7)
≥2 symptoms	53.4% (175)	46.9% (137)	44.2% (115)
≥3 symptoms	39.6% (130)	37.3% (109)	35.0% (91)
≥4 symptoms	31.1% (102)	29.5% (86)	28.1% (73)
2023 study			
N	505	470	444
mean (SD)	1.8 (2.6)	1.5 (2.7)	1.3 (2.3)
≥2 symptoms	35.8% (181)	28.7% (135)	26.4% (117)
≥3 symptoms	25.7% (130)	20.0% (94)	18.2% (81)
≥4 symptoms	16.8% (85)	14.0% (66)	12.6% (56)

**Table 3:** Estimated logistic model for long COVID using the 2022 study wave data.

	Log Odds Ratio (SE)	*p* Value	Odds Ratio
No up-to-date vaccination	0.54 (0.20)	0.006	1.72 (1.17, 2.54)
Age group, years			
18–29	Reference	-	-
30–49	0.21 (0.21)	0.331	1.23 (0.81, 1.86)
50–64	0.54 (0.26)	0.034	1.72 (1.04, 2.84)
65+	0.02 (0.36)	0.954	1.02 (0.50, 2.07)
Female	0.72 (0.20)	<0.001	2.06 (1.38, 3.08)
Index day number of acute symptoms	0.52 (0.05)	<0.001	1.69 (1.54, 1.86)
Week 1 change in number of acute symptoms	0.40 (0.04)	<0.001	1.49 (1.37, 1.63)
Number of comorbidities ^a^	0.28 (0.14)	0.046	1.33 (1.01, 1.76)
Asthma or chronic lung disease	0.67 (0.31)	0.031	1.96 (1.07, 3.61)

SE = Standard Error. ^a^ Comorbidities included asthma or chronic lung disease, cirrhosis of the liver, immunocompromised conditions or weakened immune system, diabetes, heart conditions or hypertension, and overweight or obesity.

**Table 4:** Model performance by study year, and cut-off for defining long COVID: c-statistics and 95% confidence intervals.

Cut-Off Defining Long COVID	Study Year	
	2022	2023
≥3 symptoms	0.79 (0.76, 0.82)	0.76 (0.73, 0.79)
≥2 symptoms	0.76 (0.73, 0.79)	0.73 (0.71, 0.76)
≥4 symptoms	0.83 (0.80, 0.86)	0.77 (0.74, 0.80)

## Data Availability

Aggregated data that support the findings of this study are available upon reasonable request from the corresponding author X.S., subject to review. These data are not publicly available due to them containing information that could compromise research participant privacy/consent.

## Supplementary Materials

The following supporting information can be downloaded at: https://www.mdpi.com/article/10.3390/healthcare12232321/s1. Table S1. Summary of self-reported comorbidities and acute respiratory infection symptoms; Table S2. Logistic regression model based on stepwise variable selection using 2022 study; and Figure S1. Frequency of long COVID.

## Abbreviations

CDC	The Centers for Disease Control and Prevention
COVID-19	Coronavirus Disease 2019
OR	odds ratio
SD	standard deviation
SE	standard error
SVI	social vulnerability index
