# Supplementary material for: Predictors of Long COVID Among Symptomatic US Adults Testing Positive for SARS-CoV-2 at a National Retail Pharmacy
Source: Healthcare (Basel). 2024 Nov 21;12(23):2321. doi: 10.3390/healthcare12232321 (PMC11641684; doi:10.3390/healthcare12232321)
Supplement: Supplementary file 1 [file healthcare-12-02321-s001.zip › healthcare-3288358-supplementary.pdf]

Predictors of Long COVID Among Symptomatic US Adults Testing Positive for SARS-CoV-2 at a National Retail Pharmacy

Table S1. Summary of self-reported comorbidities and acute respiratory infection symptoms

	**Year 2022 (n=328)**	**Year 2023 (n=505)**	**p-value [a]**
Self-reported comorbidities			
Asthma or chronic lung disease	30 (9.2%)	25 (5.0%)	0.017
Cirrhosis of the liver	1 (0.3%)	0 (0.0%)	0.214
Immunocompromised conditions or weakened immune system	16 (4.9%)	2 (0.4%)	0.000
Diabetes	11 (3.4%)	27 (5.4%)	0.178
Heart conditions or hypertension	41 (12.5%)	79 (15.6%)	0.207
Overweight or obesity	16 (4.9%)	60 (11.9%)	0.001
Anti-viral treatment	-	122 (24.2%)	
Symptoms on the day of SARS-CoV-2 testing			
Systemic symptoms	285 (86.9%)	451 (89.3%)	0.288
Fever	127 (38.7%)	205 (40.6%)	0.589
Chills	165 (50.3%)	255 (50.5%)	0.957
Muscle or Body Aches	183 (55.8%)	241 (47.7%)	0.023
Headache	224 (68.3%)	319 (63.2%)	0.129
Fatigue	204 (62.2%)	331 (65.5%)	0.325
Respiratory symptoms	313 (95.4%)	492 (97.4%)	0.118
Shortness of Breath or Difficulty Breathing	42 (12.8%)	75 (14.9%)	0.406
Cough	243 (74.1%)	387 (76.6%)	0.403
Sore Throat	187 (57.0%)	312 (61.8%)	0.170
New/Recent Loss of Taste or Smell	35 (10.7%)	69 (13.7%)	0.202
Congestion or Runny Nose	247 (75.3%)	410 (81.2%)	0.042
GI symptoms	93 (28.4%)	69 (13.7%)	<0.001
Nausea or Vomiting	42 (12.8%)	20 (4.0%)	<0.001
Diarrhea	69 (21.0%)	59 (11.7%)	<0.001
Symptoms at Week 1 after testing			
Systemic symptoms	202 (61.6%)	301 (59.6%)	0.568
Fever	11 (3.4%)	15 (3.0%)	0.756
Chills	20 (6.1%)	20 (4.0%)	0.159
Muscle or Body Aches	62 (18.9%)	80 (15.8%)	0.251
Headache	96 (29.3%)	119 (23.6%)	0.066
Fatigue	163 (49.7%)	259 (51.3%)	0.653
Respiratory symptoms	243 (74.1%)	373 (73.9%)	0.943
Shortness of Breath or Difficulty Breathing	69 (21.0%)	85 (16.8%)	0.127
Cough	167 (50.9%)	251 (49.7%)	0.733
Sore Throat	58 (17.7%)	66 (13.1%)	0.068
New/Recent Loss of Taste or Smell	56 (17.1%)	68 (13.5%)	0.153
Congestion or Runny Nose	154 (47.0%)	218 (43.2%)	0.283

GI symptoms	43 (13.1%)	43 (8.5%)	0.033
Nausea or Vomiting	23 (7.0%)	3 (0.6%)	<0.001
Diarrhea	26 (7.9%)	40 (7.9%)	0.998

Table S2. Logistic regression model based on stepwise variable selection using 2022 study

	**Log Odds Ratio Estimate (SE)**	**P-value**
Age	0.012 (0.006)	0.047
Female	0.781 (0.206)	<0.001
No up-to-date vaccination	0.603 (0.200)	0.003
Number of acute symptoms on the day of SARS-CoV-2 testing	0.510 (0.056)	<.0001
Increase in number of acute symptoms from the day of SARS-CoV-2 testing to Week 1	0.417 (0.044)	<.0001
Nausea or vomiting	0.807 (0.262)	0.002
Cough	-0.453 (0.212)	0.032
Congestion	0.430 (0.214)	0.044
Number of comorbidities	0.284 (0.144)	0.048
Asthma or chronic lung disease	0.683 (0.312)	0.029

SE = Standard Error

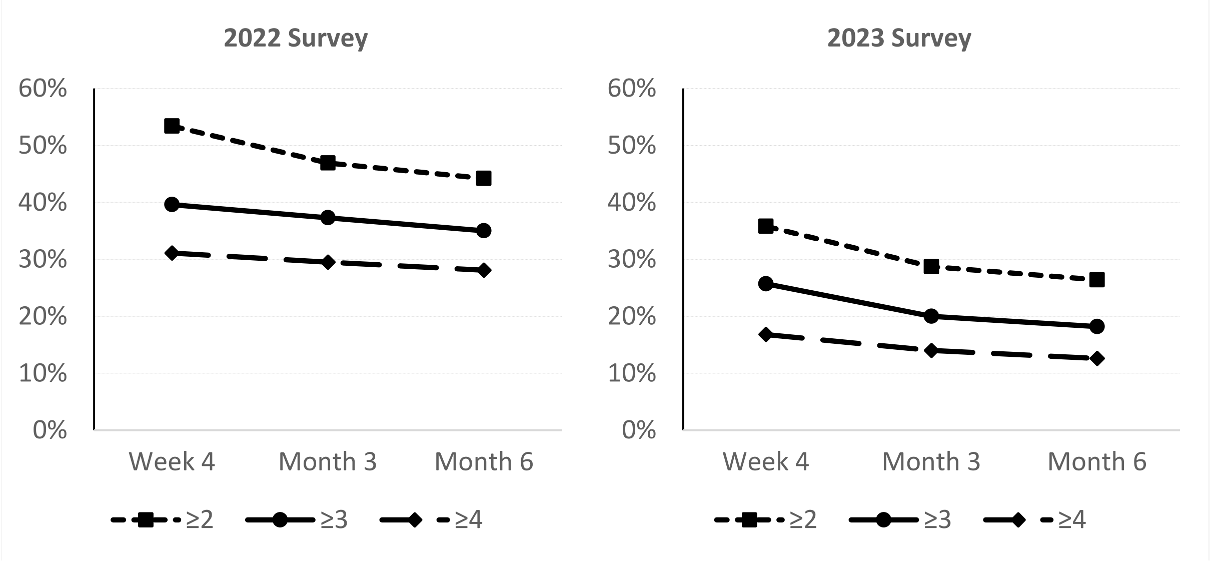

Figure S1. Frequency of long COVID
